# Orthodontic Aligners: Current Perspectives for the Modern Orthodontic Office

**DOI:** 10.3390/medicina59101773

**Published:** 2023-10-05

**Authors:** Chung How Kau, Jen Soh, Teti Christou, Akanksha Mangal

**Affiliations:** 1 Department of Orthodontics, University of Alabama at Birmingham, Birmingham, AL 35233, USA; tetich@uab.edu; 2 Department of Surgery, Heersink School of Medicine, University of Alabama at Birmingham, Birmingham, AL 35233, USA; 3 Private Orthodontic Practice, Braces & Aesthetics Dental Clinic Pte Ltd., Singapore 307506, Singapore; sohjen69@yahoo.com; 4 Orthodontic Clinical Fellowship, School of Orthodontics, Jacksonville University, Jacksonville, FL 32211, USA; akanksha.mngl@gmail.com

**Keywords:** orthodontic aligners, aligner biomechanics, innovations

## Abstract

Orthodontic aligners are changing the practice of orthodontics. This system of orthodontic appliances is becoming the mainstay appliance of choice for orthodontic offices in many countries. Patient preferences and lifestyle needs have made this appliance the primary choice when seeking care. In the early days, appliances lacked the efficiency and effectiveness of traditional bracket-wire systems, but modern systems are now able to handle a more comprehensive orthodontic caseload. Current systems provide newer biomechanical strategies and artificial intelligence-driven tooth movements for better outcomes. These improvements now mean that an orthodontist can be better prepared to manage a larger number of orthodontic malocclusions. This paper aims to discuss some of the evolution of orthodontic aligners and to describe to orthodontists the fundamentals of aligner therapy. In addition, it will provide an evidence-based outcome to the existing treatment outcomes in the current literature.

## 1. Introduction

Orthodontic aligner therapy (OAT) is changing the dental and orthodontic landscape [1]. Recent reports suggest that OAT now represents 30–45% of an orthodontic practice caseload, and there are shifting demographics on patient preferences and awareness of OAT [2,3,4]. Early orthodontic appliances certainly had many reported shortcomings [5,6], but the modern versions of OAT have had many improvements and better outcomes [7,8,9]. As a result of this success, many orthodontic practices have had to re-invent their practices to meet patient needs and alter clinical workflows [10].

In order for the modern appliances to be understood, a short history of appliance inception must be understood. The first minor tooth movement appliance was introduced by Remensnyder in 1925. This appliance, the Flex O-Tite, was used primarily to treat periodontitis. This was followed by Kesling, who introduced the “tooth positioning appliance” in 1945 [11]. In 1959, Nahoum built on Kesling’s idea of using a series of positioners to achieve tooth movements and introduced the first documented clear thermoplastic appliance, which was vacuum-based and showed a firm adaptation to the patient’s cast. This appliance could also be used as a retainer, splint, surgical pack holder, medicament carrier, etc. He also used auxiliary elements, like acrylic buttons for interarch elastics, a concept that is similar to the use of attachments and auxiliaries in the current practice of clear aligner therapy, suggesting that it may have originated from Nahoum’s treatment mechanics [12]. Later, in the 1970s, Ponitz proposed an “Invisible retainer” that was initially fabricated for finishing and retaining orthodontic cases [13]. In 1985, McNamara modified the vacuum form technique developed by Ponitz and used a Biostar machine that applied positive air pressure to fabricate an invisible retainer using a 1 mm thick Biocryl sheet, which could also be used for final detailing and retention [14]. In 1993, Sheridan further modified it by reducing the thickness to 0.030 and introduced the “Essix appliance” [12].

The core principle in OAT is the determination of the final position of all of the teeth. This philosophy of beginning with the “end in mind” was tedious and time-consuming when the concept was first proposed. The anticipated final position of the teeth was determined by sectioning the teeth on a dental cast and manipulating their positions to create tooth movement. Through trial and error, an adequate amount of tooth movement in six degrees of freedom was prescribed and achieved. However, in 1997, two Stanford graduates, Zia Chishti and Kelsey Wirth, along with two orthodontists, founded Align Technology (Santa Clara, CA, USA) [15]. Essentially, the first patents that were written described a computerized method to move teeth, through a series of polymeric shells, from a start point to a final result or end point. This revolutionary method produced the first orthodontic appliance fabricated using transparent and thermoplastic polymeric materials, with the aid of modern CAD/CAM technology. This manufacturing workflow meant that the original methods involving the manual fabrication of clear aligners could be discarded and that mass production workflows could be created. Today, the early manual methods are no longer used for commercial aligner fabrication, and the current digital fabrication method employs CAD/CAM technology and a digital workflow protocol [16,17,18,19,20]. In many ways, Align Technology must be credited with the continuous innovation and research in enhancing tooth movement and biomechanics in orthodontic aligner therapy (OAT) for both the clinician and patient [21]. Since its inception, Invisalign appliances have evolved drastically from the first-generation aligners to the present-day eight-generation aligners. During this evolutionary process, the main progress has been seen in the types of material from Proceed 30 (a polymer mixture) to Smart Track material (multi-layer aromatic thermoplastic polyurethane/co-polyester material). In addition, “SmartForce” features allow for the addition of different types of auxiliaries and attachments to the clinical software simulation. Furthermore, significant improvements to the software now allow for the incorporation of CBCT scans into the ClinCheck software, “SmartStage”, for extraction cases [22], “Invisalign first” for mixed dentition malocclusions [23], and mandibular advancement for Skeletal Class II correction, which all aim to provide greater control for tooth movements [24]. These tools also help clinicians communicate effectively to patients and also visualize the clinical treatment plans. At present, Invisalign has been the most popular to date and continues to be a leader in the market in a large pool of companies all across the world.

## 2. Beginning with the End in Mind

**Figure 1:**
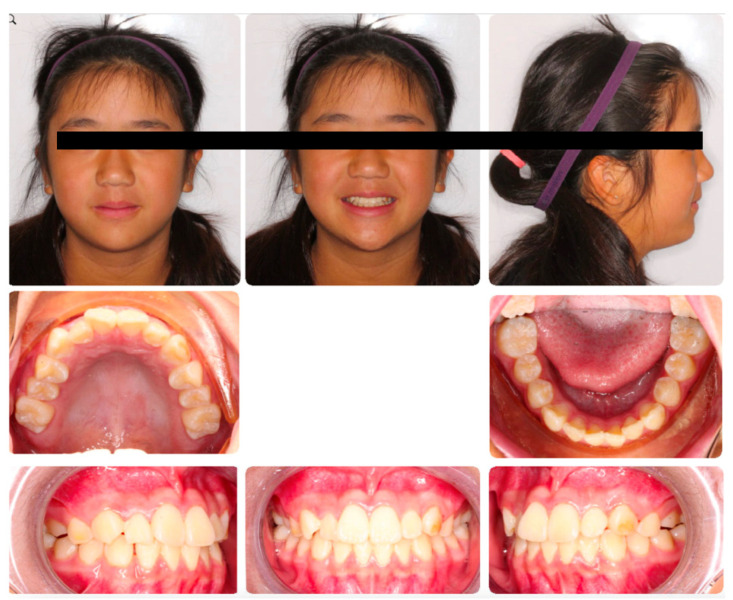
Standard array of pictures for the capture of malocclusion in an 11-year-old patient.

**Figure 2:**
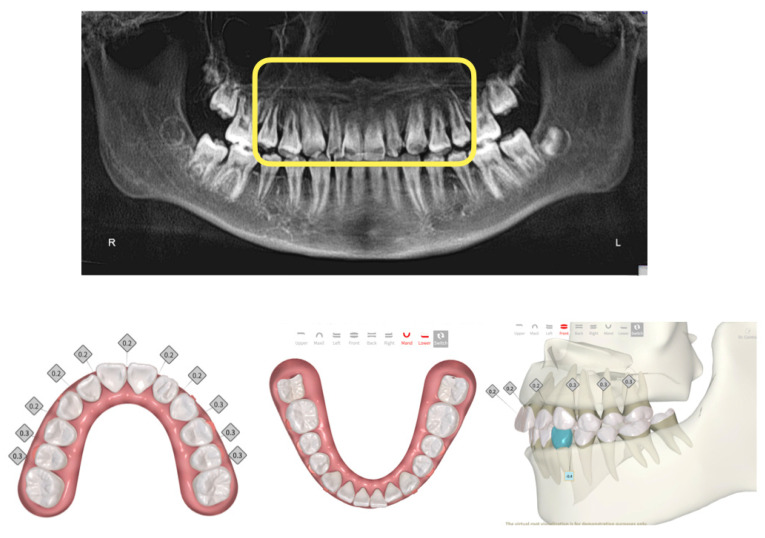
CBCT data show short roots in the anterior zone of the maxillary teeth. Careful treatment planning with the Smartee check clinical simulation software reduced the overall rate of tooth movement in the patient’s treatment plan and also allowed for visualization of the roots. Total number of trays was 30 in this treatment sequence.

**Figure 3:**
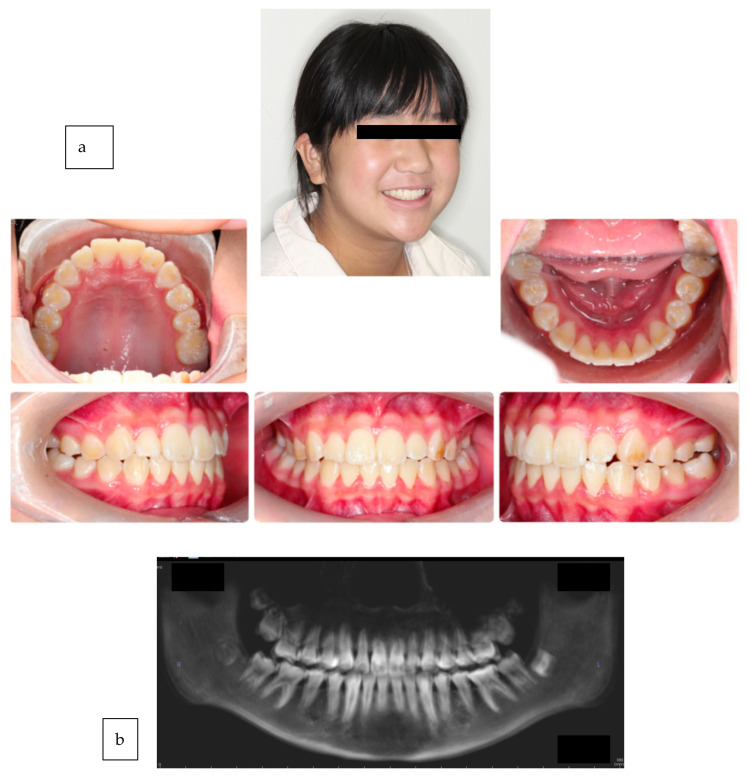
(**a**,**b**) Completion of 30 sets of trays in under 24 weeks. No further root resorption to the anterior teeth and a highly desirable esthetic result for maximum smile projection.

Many OAT methods allow for the visualization of the patient end result. This key treatment planning feature now allows for many clinicians to enhance their treatment plans and optimize smile aesthetics [25]. Recent publications showed that finished treatments presented to the American Board of Orthodontics still required significant improvements when comparing Invisalign and traditional fixed appliance therapy despite the advantage of having the ClinCheck visualization software tool that predicts the simulated final outcome of the treated occlusion [26,27]. The ability to visualize the final result in conjunction with the use of cone beam computed tomography has expanded the diagnostic ability to study the position of roots, apical root resorption, and bony changes associated with the planned tooth movements (Figure 1, Figure 2 and Figure 3) [28,29].

## 3. Clinical Effectiveness of Orthodontic Aligner Therapy

**Figure 4:**
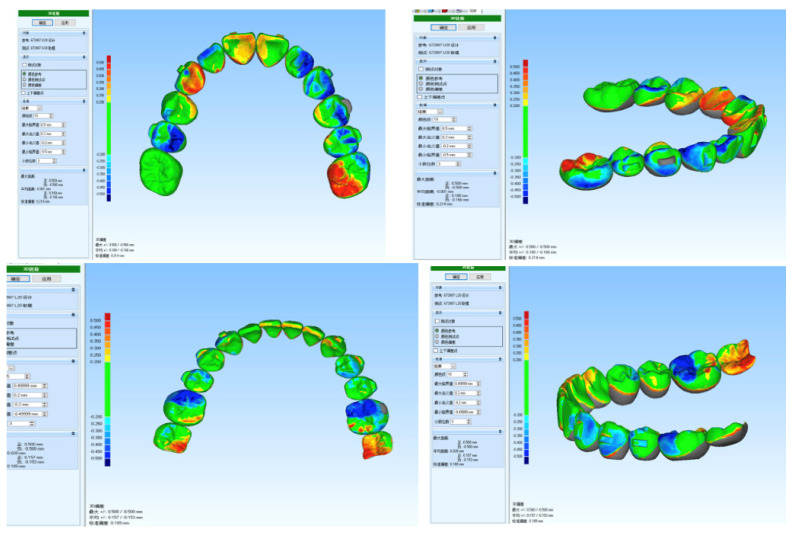
Superimposition of Smartee check tooth simulated results with real intra-oral patient scan data. The results of the patient at T30 were less than 0.25 mm across all tooth movements. This showed that the planned clinical result is producing the desired tooth movement and is one way to ensure proper tracking of the aligners during the patient’s treatment.

Although the range of treatment indications for OAT has broadened over time, the predictability of all movements planned in the main phase of treatment does not reach anywhere near 100%. The average accuracy of OAT ranged between 41% and 73%, depending mainly on the type of movement, its magnitude, and the tooth type investigated [30,31]. Simple studies to understand the “tracking” outcomes of clinically planned simulations and actual tooth movement will help in understanding the effectiveness of OAT (Figure 4). A number of studies have also described problematic movements. These include root torque, bodily translation [31], and the derotation of round-shaped teeth [32], which often entail mid-course corrections and/or additional finishing phases. Extrusion and intrusion mechanics have also been challenges, but clinicians have overcome some of these shortfalls by changing the pathways in which these teeth move on the computer simulations. Table 1 describes some of the movements reported in the literature. Many clinicians have also described the importance of the type of aligner and the material the aligner is made from, not to mention the planning accuracy, the manual skill of the orthodontist, and patient compliance [32].

## 4. Materials

Orthodontic aligners have undergone multiple transformations over the years. The need for improved clinical efficiency to treat various malocclusions has increased, so there has been an evolution in clinical materials. Every new aligner manufacturer claims to be better than the previous one. Many manufacturers claim that various factors like the material type, the thickness of the plastic, and its mechanical properties influence the effectiveness and efficiency of the ultimate aligner produced. The first generation of aligners used a polymer mixture called Proceed 30. Its main aim was to treat mild crowding and close spaces in simple cases [34]. However, it did not meet all the physical, chemical, and clinical requirements for orthodontic tooth movement [35,36]. These early issues led to modifications and advances in aligner systems. These changes included the addition of attachments for better control of the tooth movement, and changes in the material that was used to produce aligners, aiming to incorporate the properties that are essential to achieve the predicted orthodontic tooth movement. “Exceed 30” was a single-layer polymer material that provided 1.5 times greater elasticity and 4 times more adaptability than Proceed 30. It was also more patient-friendly in terms of the insertion and removal of the aligners. The pursuit to improve the aligner material by improving the aligner material properties continues to this day. In 2013, the Exceed 30 material was replaced by a new multi-layer material called the SmartTrack ^TM^ (LD30). This material was very superior to its precursors as it performed better. It delivered consistent forces that were gentle and had long-term action, and it had improved properties like greater elasticity, chemical stability, and a precise fit [37,38,39,40]. It was also more comfortable to patients [41]. Since then, developments in the aligner systems have been involved with increasing the ability to treat more complex and severe malocclusions compared to the ones that can be treated successfully with fixed orthodontic treatments.

The latest trend in the development of OAT is shape-memory polymers (SMPs) and direct 3D printing of the aligners [42,43,44]. SMPs are a type of smart material or stimuli-responsive polymer material. These materials are able to react suitably with external stimuli, such as thermal, electrical, or magnetic input, producing a predictable repeatable output. SMPs have the ability to change their macroscopic shape under a proper stimulus. Some preliminary in vitro investigations using a thermal responsive SMP (shape-memory sheet (ClearX)) by Kline Europe GmbH, Düsseldorf, Germany and Graphy material have shown tremendous promise [44]. Unlike shape-changing materials (conventional materials), shape-memory materials have the capacity to maintain a stable temporary shape until they are appropriately activated to recover their original shape. Shape recovery forces generated upon appropriate thermal stimuli were used to move a tooth on a typodont. The aim was to overcome the rate-limiting staging of conventional aligner materials and show the possibility of using one shape-memory aligner instead of three subsequent conventional aligners in order to decrease the number of aligners used per treatment, saving money and time, reducing plastic consumption, and consequently decreasing the total cost. One added advantage was that thermo-responsive SMPs were transparent and aesthetically pleasing. This, along with their intrinsic shape recovery property, made them a novel orthodontic material. Elshazly and co-authors concluded that experimentally, tooth movement could be conducted on a typodont model by using clear aligners made of shape-memory polymers (SMPs) [45]. The aligner, however, should undergo different steps of special heat treatment above its transition temperature in order to initiate its shape-memory recovery. Aligners made of SMPs could be a promising future choice for aesthetic orthodontic treatment [46].

**Figure 5:**
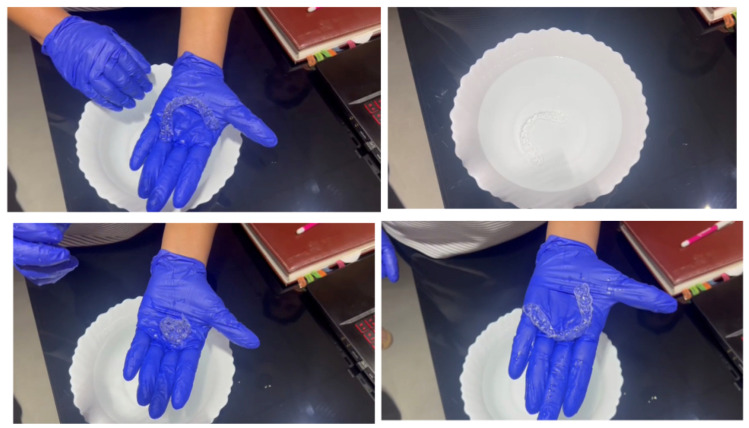
A direct 3D-printed aligner being placed in a water bath of 35 degrees Celsius. Notice that the aligner is now able to deform under pressure but recovers its shape memory when placed back into the water bath. This is an important first step in direct 3D-printed aligners.

Just as digital impressions and 3D-printed models proved superior to manual impressions and plaster models, clear aligners that are 3D printed directly may eliminate the errors resulting from the thermoplastic workflow, apart from the errors that result from analog impressions [14]. In September 2021, the South Korean manufacturer, Graphy, showcased the world’s first direct 3D-printed aligner, which was produced from the company’s own 3D printing resin. This resin is a patented technology called Tera Harz TC-35, which is a clear biocompatible photocurable resin that claims to be equipped with a shape-memory function that, according to Graphy, is the only one available in the current market [43]. Graphy also claimed that these aligners could be produced from any 3D printer. Another feature includes the ability of the aligner to rotate teeth by up to 35°, which other aligners may struggle to achieve. Compared to the manufacturing method of existing transparent aligners, model output is not required, and manual work such as thermoforming, cutting, and finishing is not required, so there are few errors and less waste during the manufacturing process, and the manufacturing time and cost are also reduced. Moreover, heat sterilization is possible with this material. In the case of thermoforming materials, they are completely deformed in hot water, whereas in the case of the material used by Graphy, their shape and physical properties are restored in hot water, so if the device is used for a long time or if the device is contaminated by food or foreign substances, it can be heat-sterilized by putting it in hot water and can always be kept clean (Figure 5). An in vitro study found that changing the thickness of a direct 3D-printed aligner (TC-35) resulted in changes to the magnitude of forces and moments generated that were more optimized with minimal unwanted side effects, thereby producing more predictable tooth movement [42]. Yet another study found that a direct 3D-printed aligner (TC-35) was able to achieve higher extrusive forces on the maxillary central incisor without attachments by using pressure columns in the aligner itself when compared with thermoformed aligners [47]. Finally, a direct 3D-printed aligner was found to deliver more consistent forces with fewer unwanted side effects over a 14-day period in an in vitro study [43]. The development of this material is revolutionary as it incorporates the benefits of shape memory and 3D printing in one aligner system.

## 5. Commercial Aligner Companies

Align Technology is the pioneering parent company of Invisalign that offers commercially produced clear aligners. The interactive software for tooth movement planning is known as ClinCheck. Invisalign provides a clinical guide on the complexity of malocclusion that governs the choice of the different delivery packages based on the number of aligners that are predicted by the ClinCheck software that simulates the OAT outcomes. The packages comprise Express, Lite, Moderate, and Comprehensive, with an increasing number of aligners for each respective package to treat the increasing complexity of the malocclusion.

Besides Align Technology, other recent commercial companies that produce clear aligners include Henry Schein (Reveal), Straumann (Clear Correct), Angel Align, Ormco (Spark), and Smartee, to name a few. Each company has its proprietary interactive tooth movement planning software for communication between the clinician and the technician, assisting with the tooth movement planning process.

Many companies incorporate clinical teeth that allow for the integration of 3D CBCT data into the crowns of the teeth. These revolutionary changes now mean that a true 3D virtual patient may be acquired for diagnosis, treatment planning, and appliance fabrication [28,29].

## 6. OAT Differentiations

As clinicians make choices for their dental offices, how would one differentiate the vast number of OAT appliance manufacturers? This paper proposes a simple method to evaluate the various OAT appliance manufacturers. Table 2 describes the various decisions that a clinician can make when investing in a particular system. Not all practices are ready to embrace a fully integrated orthodontic office, and enhanced decisions have to be made based on patient population, practice need, investment in the practice, and brand positioning.

## 7. Direct-to-Consumer (DTC) Aligners

Direct-to-Consumer (DTC) aligners pose a challenge to the modern orthodontic office that offers CAT in that the delivery of aligners is directly from the commercial companies to the patients. A recent modification to the original modus operandi of some of these DTC companies was the enlistment of clinicians from participating dental clinics to conduct a general clinical and radiographic dental examination for caries and periodontal problems, place attachments, and issue aligners and to perform an interproximal reduction based on the instructions dictated by the treatment plan developed by the companies. The drawback of DTC aligners is the lack of clinical ownership, as the participating clinician is not the one who assesses and plans the tooth movement; it is performed remotely by the clinical team that is employed by the DTC companies. The modern orthodontic office must be prepared to manage and treat failures of DTC aligners. Thus, a modern orthodontic office must be equipped not only to deliver in-office OAT, but also to educate patients on the differences between in-office OAT and DTC aligners [48,49,50].

## 8. Clinical Performance of Orthodontic Clear Aligners

A prospective study conducted in 2009 found that the mean accuracy of tooth movement with Invisalign aligners was at best 41%. Lingual constriction was found to be the most accurate at 47.1%. The least accurate movements were the extrusion of maxillary and mandibular incisors, followed by the mesiodistal tipping of canines. A rotational movement greater than 15 degrees resulted in a decrease in the accuracy of the tooth movement that was simulated [51]. A similar prospective study conducted in 2020 found that the mean accuracy had improved to 50% for all tooth movements. Although the overall accuracy had improved, the study concluded that the strengths and weaknesses of Invisalign aligners did not change much over the years of its research and development [33].

A systematic review concluded that Invisalign was a viable tool to treat mild to moderate malocclusions in non-growing patients with a non-extraction approach. Clear recommendations for other treatment strategies with the use of aligners could not be ascertained due to a lack of standardized protocols and high variations of clinical and research methods. Limited efficacy was found for arch expansion with bodily tooth movement, the closure of extraction spaces, corrections of greater vertical and anteroposterior discrepancies, and establishing occlusal contacts [52]. Another systematic review found a low-to-moderate level of evidence for tooth movement efficacy in orthodontic aligner therapy. Acceptable outcomes were comparable with fixed appliance therapy for mild to moderate malocclusions. The review also concluded that predictable tooth movements could not be achieved with a single series of aligners [53].

A retrospective clinical study found that one in every six patients who started with OAT was converted to fixed appliance therapy to complete the treatment. Patients with an average number of 81 aligners were more likely to switch and complete the treatment with a fixed appliance. A longer overall treatment time was demonstrated in patients who converted to a fixed appliance. Only 6% of OAT patients completed their treatment without additional refinement aligners. The study also concluded that two to three additional series of refinement aligners could be expected, and the average treatment time was 2 years [54].

An unwanted effect associated with OAT is unplanned molar intrusion due to the interface between the upper and lower aligner materials contacting each other. A study found that 74% of patients demonstrated unplanned molar intrusion after treatment. Maxillary molar intrusion accounted for 15.5%, and mandibular molar intrusion accounted for 32.8%, while maxillary and mandibular molar intrusions accounted for 25.9%. A vertical facial type and muscular stimulus while wearing aligners were proposed as possible factors associated with this unplanned effect. Although the investigators were of the opinion that unplanned molar intrusion was self-limiting, vertical occlusal settling and spontaneous re-equilibration over a longer period of time were not evaluated and would require further investigation and validation [55].

In summary, the recent research studies suggest that the efficacy of aligners has yet to achieve a high level of predictability for the full range of tooth movements. This would impact the overall treatment duration when the predicted number of aligners needed to deliver the outcome is not matched clinically. Greater amounts of clinical time and effort are needed with either an additional series of refinement aligners or a conversion to a fixed appliance in order to finish and detail the occlusion to achieve the required treatment outcome.

## 9. Future Research

The recent research on orthodontic aligner therapy has provided a better understanding of plastic material behavior and tooth movement responses. However, much research is needed to provide evidence-based clinical data for the following:

1. Factors that determine the choice of 7-, 10-, or 14-day protocols for an aligner change.
2. The in vivo performance of optimized versus conventional attachments for all types of tooth movements besides rotation and extrusion.
3. The accuracy of the amount of interproximal reduction in the enamel determined by the software to facilitate the required tooth movement that will be clinically achieved.
4. The magnitude and degree of overcorrection for the various types of tooth movements in order to compensate for the inherent limitation of the plastic material to fully express the desired tooth movement or to counteract unwanted tooth movement.
5. The mid- and long-term outcomes of unplanned molar intrusion on the vertical settling of functional occlusion during retention.
6. An improvement in the predictability of the software simulations of treatment outcomes that are realistically achievable clinically.
7. The accuracy of the prediction of gingival margin height changes with tooth movement such as extrusion and intrusion.

## 10. Conclusions and Final Thoughts

The future brings tremendous promise for clinicians. The ability to customize and individualize a patient’s treatment journey is exciting yet daunting. The authors have found that the early adoption of technology can sometimes be exciting, but beta versions of new technology often do not live up to their expectations [56]. The fundamentals of orthodontic examination and diagnosis, treatment planning, and biomechanics combined with clinical competency, experience, and acumen remain the pillars of sound orthodontic treatment. As a result, a careful understanding of technology coupled with clinical-based evidence is the key to success.

## Figures and Tables

**Table 1:** Table showing the range of tooth movements for various aligners and their effectiveness as reported in the literature.

Year	Author	Study Design	Malocclusion	Patient Group	Extrusion	Intrusion	Rotation	Bodily Movement	Tipping
2022	Palone et al. [30] aligner system: F22 Aligners Overall: approximately 20% (19.42%)	Retrospective need for overcorrection (measured the amount of correction required in the finishing phase to achieve the prescribed outcomes)	Class I malocclusion, minimal crowding (≤3 mm)	150 patients (80 women and 70 men; mean age: 33.7 years)	11.7%	22%	Greatest correction necessary - 40.2% upper incisors - 39.7% lower incisors - 28.8% upper canines - 28.7% lower canines		20.5% inclination (buccal–lingual crown tipping) 14.5% angulation (mesial–distal crown tipping)
2019	Haouili et al. [33] aligner system: Invisalign Mean accuracy for all TMs: 50%	Prospective follow-up study	22 Class I, 13 Class II, and 3 Class III	38 patients Mean age: 36 years	Extrusion of the maxillary incisors (55%) had the highest accuracy, whereas extrusion of the maxillary and mandibular molars (40%) had the lowest accuracy	*Incisor intrusion remained a challenge* *And did not improve (35%),* whereas second molar intrusion had high accuracy (51%)	*Lowest overall accuracy (46%)* - Difficult for canines, premolars, and molars - Distal rotation (37%) was significantly less accurate than mesial rotation (52%) - Mesial rotation of max 1st molars (28%—least accurate)		Buccal–lingual crown tip was *overall most accurate (56%) tooth movement* Labial crown tip max LI (70%)—most accurate
2019	Jiang et al. [31] aligner system: Invisalign	Retrospective measurement of different types of incisor movements in sagittal plane	Crowding Non-extraction	69 patients Age ≥ 20 years Overall efficacy: 55.58%				Translation: 49.50% - Less effective on maxillary incisors compared to mandibular incisors	- Highest accuracy: pure tipping (77.48%) - Controlled tipping: 65.24% - Torque: 35.21% (lingual root movement was significantly more difficult to accomplish than labial root movement)
2018	Charalampakis et al. [34] aligner system: Invisalign	Retrospective	Class I (mild, mod, severe) - Crowding - Deep bite - Open bite	20 adult patients Avg age: 37 years and 6 months	- Accurate - No statistically significant differences observed	- Most inaccurate of all linear movements - Differences from 0.8 to 1.5 mm - The maxillary central incisors had the greatest difference of 1.5 mm	- Most inaccurate - Discrepancy of 0.9 to 3.06 degrees - Highest inaccuracy in the maxillary canine region		Horizontal movements of all incisors (buccal-lingual crown tip): accurate, with either small (0.20–0.25 mm) or insignificant differences
2015	Rossini et al. [32] aligner system: Invisalign	Systematic review			- Not effective in controlling anterior extrusion - Contrasting results for posterior vertical control, so a definite conclusion cannot be drawn	Anterior intrusion is achievable with OAT and is comparable to that reported for the straight wire technique	OAT is not effective in controlling rotations, especially for rounded teeth	Orthodontic Aligner Therapy (OAT) is effective in controlling upper molar bodily movement when a distalization of 1.5 mm is prescribed	

**Table 2:** A simple table that any clinician can use when evaluating the aligner manufacturer of choice for the dental office.

		Comprehensive	Normal	Basic	Limited
Patient Records	Clinical Pictures and Radiographs	X	X	X	
Intra-Oral Scan Uploads / PVS Impressions	Dedicated	X	X		
	3rd Party			X	X
Software Interface	Interactive	X	X		
	Static			X	X
Tooth Movement	All Teeth	X	X		
	Most Teeth			X	
	Some Teeth				X
Attachments	All Teeth	X	X	X	
3D Controls	Real-Time Movements	X	X	X	
	Real-Time Attachments	X	X		
	Real-Time IPR	X			
	Real-Time Simulation of Doctor Changes	X			
	Tray Cut-Outs	X			
Complex Tooth Movements	Tooth Expansion	X			
	Sagittal Correctors	X			
Interactive Patient Tools	Predictive Simulation	X	X		
	Compliance Indicators Built Into Aligners	X			
Software	Subscription Fees	X	X	X	X
Clinical Research		X	X		
Post Treatment	Provision of Invisible Retainers	X	X		
	Duration of Coverage of Aligner Production Fees (Clearly Stated Fees)	X	X		

X—needed requirement.

## References

[1] Hartshorne J., Wertheimer M.B. (2022). Emerging insights and new developments in clear aligner therapy: A review of the literature. AJO-DO Clin. Companion.

[2] Abu-Arqub S., Ahmida A., Da Cunha Godoy L., Kuo C.L., Upadhyay M., Yadav S. (2023). Insight into clear aligner therapy protocols and preferences among members of the American Association of Orthodontists in the United States and Canada. Angle Orthod..

[3] Baxmann M., Timm L.H., Schwendicke F. (2022). Who Seeks Clear Aligner Therapy? A European Cross-National Real-World Data Analysis. Life.

[4] Best A.D., Shroff B., Carrico C.K., Lindauer S.J. (2017). Treatment management between orthodontists and general practitioners performing clear aligner therapy. Angle Orthod..

[5] Wheeler T. (2005). Invisalign clinical trials needed. Am. J. Orthod. Dentofac. Orthop..

[6] McKenna S. (2001). Invisalign: Technology or mythology?. J. Mass. Dent. Soc..

[7] Meade M.J., Ng E., Weir T. (2023). Digital treatment planning and clear aligner therapy: A retrospective cohort study. J. Orthod..

[8] Miller K.B., McGorray S.P., Womack R., Quintero J.C., Perelmuter M., Gibson J., Dolan T.A., Wheeler T.T. (2007). A comparison of treatment impacts between Invisalign aligner and fixed appliance therapy during the first week of treatment. Am. J. Orthod. Dentofac. Orthop..

[9] Kau C.H., Feinberg K.B., Christou T. (2017). Effectiveness of Clear Aligners in Treating Patients with Anterior Open Bite: A Retrospective Analysis. J. Clin. Orthod..

[10] Zhao Z.H. (2019). Clear aligner therapy: Risks and clinical strategies. Zhonghua Kou Qiang Yi Xue Za Zhi.

[11] Kesling H.D. (1946). Coordinating the predetermined pattern and tooth positioner with conventional treatment. Am. J. Orthod. Oral Surg..

[12] Sheridan J.J., McMinn R., LeDoux W. (1995). Essix thermosealed appliances: Various orthodontic uses. J. Clin. Orthod..

[13] Ponitz R.J. (1971). Invisible retainers. Am. J. Orthod..

[14] McNamara J.A., Kramer K.L., Juenker J.P. (1985). Invisible retainers. J. Clin. Orthod..

[15] Vlaskalic V., Boyd R.L. (2002). Clinical evolution of the Invisalign appliance. J. Calif. Dent. Assoc..

[16] Gierie W.V. (2018). Clear aligner therapy: An overview. J. Clin. Orthod..

[17] Lim Z.W., Meade M.J., Weir T. (2023). The predictability of maxillary curve of Spee leveling with the Invisalign appliance. Angle Orthod..

[18] Xie J., Liu F., Sang T., Wu J. (2023). Factors affecting the efficacy of Invisalign in anterior tooth rotation. Am. J. Orthod. Dentofac. Orthop..

[19] Tien R., Patel V., Chen T., Lavrin I., Naoum S., Lee R.J., Goonewardene M.S. (2023). The predictability of expansion with Invisalign: A retrospective cohort study. Am. J. Orthod. Dentofac. Orthop..

[20] Kumar M., Goyal M., Kaur A. (2021). Has Invisalign improved?. Am. J. Orthod. Dentofac. Orthop..

[21] Ma S., Wang Y. (2023). Clinical outcomes of arch expansion with Invisalign: A systematic review. BMC Oral Health.

[22] Ojima K., Dan C., Nishiyama R., Ohtsuka S., Schupp W. (2014). Accelerated extraction treatment with Invisalign. J. Clin. Orthod..

[23] Wang J., Bukhari A., Tai S.K., Zou B. (2023). Dimensional changes in the palate associated with Invisalign First System: A pilot study. Angle Orthod..

[24] Lucchese A., Nocini R., Tacchino U., Ghislanzoni L.H., Bertossi D., Ricciardi G., Bassani L., Korolija S., Giudice A.L., Croce S. (2020). Invisalign appliance: Aesthetic and efficiency. Minerva Stomatol..

[25] Kau C.H., Christou T., Sharma S. (2022). Contemporary Smile Design: An Orthodontic Perspective. Dent. Clin. N. Am..

[26] Djeu G., Shelton C., Maganzini A. (2005). Outcome assessment of Invisalign and traditional orthodontic treatment compared with the American Board of Orthodontics objective grading system. Am. J. Orthod. Dentofac. Orthop..

[27] Papageorgiou S.N., Koletsi D., Iliadi A., Peltomaki T., Eliades T. (2020). Treatment outcome with orthodontic aligners and fixed appliances: A systematic review with meta-analyses. Eur. J. Orthod..

[28] Li Y., Deng S., Mei L., Li Z., Zhang X., Yang C., Li Y. (2020). Prevalence and severity of apical root resorption during orthodontic treatment with clear aligners and fixed appliances: A cone beam computed tomography study. Prog. Orthod..

[29] Santucci V., Rossouw P.E., Michelogiannakis D., El-Baily T., Feng C. (2023). Assessment of posterior dentoalveolar expansion with Invisalign in adult patients. Int. J. Environ. Res. Public Health.

[30] Palone M., Pignotti A., Morin E., Pancari C., Spedicato G.A., Cremonini F., Lombardo L. (2023). Analysis of overcorrection to be included for planning clear aligner therapy: A retrospective study. Angle Orthod..

[31] Jiang T., Jiang Y.N., Chu F.T., Lu P.J., Tang G.H. (2021). A cone-beam computed tomographic study evaluating the efficacy of incisor movement with clear aligners: Assessment of incisor pure tipping, controlled tipping, translation, and torque. Am. J. Orthod. Dentofac. Orthop..

[32] Rossini G., Parrini S., Castroflorio T., Deregibus A., Debernardi C.L. (2015). Efficacy of clear aligners in controlling orthodontic tooth movement: A systematic review. Angle Orthod..

[33] Haouili N., Kravitz N.D., Vaid N.R., Ferguson D.J., Makki L. (2020). Has Invisalign improved? A prospective follow-up study on the efficacy of tooth movement with Invisalign. Am. J. Orthod. Dentofac. Orthop..

[34] Charalampakis O., Iliadi A., Ueno H., Oliver D.R., Kim K.B. (2018). Accuracy of clear aligners: A retrospective study of patients who needed refinement. Am. J. Orthod. Dentofac. Orthop..

[35] Li B., Xu Y., Shi R., Hu Y., Liu S., Gu Z. (2022). Accuracy of progress assessment with clear aligners. Hua Xi Kou Qiang Yi Xue Za Zhi.

[36] Condo’ R., Pazzini L., Cerroni L., Pasquantonio G., Lagana’ G., Pecora A., Mussi V., Rinaldi A., Mecheri B., Licoccia S. (2018). Mechanical properties of “two generations” of teeth aligners: Change analysis during oral permanence. Dent. Mater. J..

[37] Feinberg K.B., Souccar N.M., Kau C.H., Oster R.A., Lawson N.C. (2016). Translucency, Stain Resistance, and Hardness of Composites Used for Invisalign Attachments. J. Clin. Orthod..

[38] Cremonini F., Zabini F., Oliverio T., Bianchi A., Scalia S., Siciliani G., Lombardo L. (2022). Optical properties of seven types of clear aligners before and after in vitro aging. J. Clin. Orthod..

[39] Elshazly T.M., Nang D., Golkhani B., Elattar H., Keilig L., Bourauel C. (2023). Effect of thermomechanical aging of orthodontic aligners on force and torque generation: An in vitro study. J. Mech. Behav. Biomed. Mater..

[40] Koletsi D., Panayi N., Laspos C., Athanasiou A.E., Zinelis S., Eliades T. (2022). In vivo aging-induced surface roughness alterations of Invisalign((R)) and 3D-printed aligners. J. Orthod..

[41] Bichu Y.M., Alwafi A., Liu X., Andrews J., Ludwig B., Bichu A.Y., Zou B. (2023). Advances in orthodontic clear aligner materials. Bioact. Mater..

[42] Grant J., Foley P., Bankhead B., Miranda G., Adel S.M., Kim K.B. (2023). Forces and moments generated by 3D direct printed clear aligners of varying labial and lingual thicknesses during lingual movement of maxillary central incisor: An in vitro study. Prog. Orthod..

[43] Hertan E., McCray J., Bankhead B., Kim K.B. (2022). Force profile assessment of direct-printed aligners versus thermoformed aligners and the effects of non-engaged surface patterns. Prog. Orthod..

[44] Koenig N., Choi J.-Y., McCray J., Hayes A., Schneider P., Kim K.B. (2022). Comparison of dimensional accuracy between direct-printed and thermoformed aligners. Korean J. Orthod..

[45] Elshazly T.M., Keilig L., Alkabani Y., Ghoneima A., Abuzayda M., Talaat S., Bourauel C.P. (2021). Primary Evaluation of Shape Recovery of Orthodontic Aligners Fabricated from Shape Memory Polymer (A Typodont Study). Dent. J..

[46] Bruni A., Serra F.G., Deregibus A., Castroflorio T. (2019). Shape-Memory Polymers in Dentistry: Systematic Review and Patent Landscape Report. Materials.

[47] McKay A., McCray J., Bankhead B., Lee M.M., Miranda G., Adel S.M., Kim K.B. (2023). Forces and moments generated during extrusion of a maxillary central incisor with clear aligners: An in vitro study. BMC Oral Health.

[48] Belgal P., Mhay S., Patel V., Nalliah R.P. (2023). Adverse Events Related to Direct-To-Consumer Sequential Aligners—A Study of the MAUDE Database. Dent. J..

[49] Hunsaker R.J., Shroff B., Carrico C., Alford B., Lindauer S.J. (2022). A comparison of patient testimonials on YouTube of the most common orthodontic treatment modalities: Braces, in-office aligners, and direct-to-consumer aligners. Am. J. Orthod. Dentofac. Orthop..

[50] Olson J.C., Shroff B., Carrico C., Boyle J., Lindauer S.J. (2020). Comparison of patient factors influencing the selection of an orthodontist, general dentist, or direct-to-consumer aligners. Am. J. Orthod. Dentofac. Orthop..

[51] Kravitz N.D., Kusnoto B., BeGole E., Obrez A., Agran B. (2009). How well does Invisalign work? A prospective clinical study evaluating the efficacy of tooth movement with Invisalign. Am. J. Orthod. Dentofac. Orthop..

[52] Papadimitriou A., Mousoulea S., Gkantidis N., Kloukos D. (2018). Clinical effectiveness of Invisalign^®^ orthodntic treatment: A systematic review. Prog. Orthod..

[53] Robertson L., Kaur H., Fagundes N.C.F., Romanyk D., Major P., Mir C.F. (2020). Effectiveness of clear aligner therapy for orthodontic treatment. Orthod. Craniofacial Res..

[54] Kravitz N.D., Dalloul B., Zaid Y.A., Shah C., Vaid N.R. (2023). What percentage of patients switch from Invisalign to braces? A retrospective study evaluating the conversion rate, number of refinement scans, and length of treatment. Am. J. Orthod. Dentofac. Orthop..

[55] Talens-Cogollos L., Vela-Hernández A., Peiró-Guijarro M.A., García-Sanz V., Montiel-Company J.M., Gandía-Franco J.L., Bellot-Arcís C., Paredes-Gallardo V. (2022). Unplanned molar intrusion after Invisalign treatment. Am. J. Orthod. Dentofac. Orthop..

[56] Kau C.H. (2016). Bench, Boundaries, and Benefits: Negotiating the Maze to a Successful Product in the United States. JDR Clin. Trans. Res..

